# An enoyl-ACP reductase inhibitor, NITD-916, expresses anti-*Mycobacterium abscessus* activity

**DOI:** 10.1128/aac.00249-25

**Published:** 2025-05-23

**Authors:** Yaping Jia, Junsheng Fan, Zhili Tan, Anqi Li, Siyuan He, Yani Lin, Juan Li, Zhemin Zhang, Bing Li, Haiqing Chu

**Affiliations:** 1 Department of Respiratory and Critical Care Medicine, Shanghai Pulmonary Hospital, School of Medicine,Tongji University 12476, Shanghai, China; 2 School of Medicine, Tongji University 481875, Shanghai, China; 3 Shanghai Key Laboratory of Tuberculosis, Shanghai Pulmonary Hospital, School of Medicine,Tongji University 12476, Shanghai, China; City St George's, University of London, London, United Kingdom

**Keywords:** *Mycobacterium abscessus*, NTM, NITD-916, enoyl-ACP reductase inhibitor, antimicrobial activity

## Abstract

Antibiotic therapy for *Mycobacterium abscessus* infections is challenging due to resistance of the organism to many clinically available antimicrobials. Here, the efficacy of NITD-916, an enoyl-ACP reductase inhibitor, in preventing *M. abscessus* growth *in vitro* and *in vivo* is demonstrated. The minimal inhibitory concentrations (MICs) of NITD-916 for 12 non-tuberculosis mycobacteria (NTM) reference strains and a collection of 194 clinical *M. abscessus* isolates were determined using the broth microdilution method. Compatibility of NITD-916 with 10 clinically important antibiotics was ascertained by checkerboard assay. The activity of NITD-916 against *M. abscessus* growing in cultured macrophages was also evaluated. Finally, the potency of NITD-916 *in vivo* was determined in a mouse model that mimicked an acute pulmonary *M. abscessus* infection. NITD-916 was bacteriostatic for *M. abscessus* replicating *in vitro,* expressing a MIC_50_ of 0.125 mg/L and a MIC_90_ of 1 mg/L against the screened clinical isolates. Furthermore, NITD-916 synergized with clarithromycin in treating 2 out of 5 subsp. *massiliense* strains. NITD-916 was active against *M. abscessus* replicating in both cultured macrophages and infected mice. The administration of 100 mg/kg NITD-916 for 14 days resulted in a 5.6 log_10_ colony-forming units (CFUs) reduction in the bacterial load in mouse lung tissue. NITD-916 is active against *M. abscessus in vitro* and *in vivo* and may be used potentially to treat *M. abscessus* diseases.

## INTRODUCTION

Non-tuberculous mycobacterium (NTM) infections are increasing globally and becoming a major public health challenge (1). The most common clinical manifestation of NTM infections is pulmonary disease, which can be caused by either slow-growing or rapid-growing (RGM) mycobacteria. *Mycobacterium abscessus* accounts for over 80% of RGM occurring especially in patients with chronic lung diseases (2, 3). The presence of *M. abscessus* in NTM patients frequently correlates with rapidly deteriorating lung function and extremely difficult-to-treat infections (3, 4). *M. abscessus* is intrinsically resistant to many antibiotic classes, rendering treatment complicated and often unpredictable (5). Despite complex drug therapy, often administered over the course of years, such infections exhibit low cure rates and high rates of mortality (6–9). Advancements in the development of antimycobacterial drugs will lead to the discovery of new treatment options.

The mycolic acid (MA) biosynthetic pathway is a promising target for developing antimycobacterial agents. MA, located in the outer layer of the mycobacterial cell wall, consists of a series of long-chain (C_77–79_) and short-chain (C_62–64_) α-alkyl fatty acids, which play essential roles in physiology and virulence (10, 11). Enoyl-ACP carrier protein reductase (InhA) is a crucial enzyme in the fatty acid elongation system (FAS-II) of mycobacteria, particularly in MA synthesis (10). Upon activation, prodrugs such as isoniazid (INH) and ethionamide (ETH) inhibit InhA in FAS-II, blocking MA synthesis and cell wall construction (12). *M. abscessus*, however, either lacks the enzymes required to activate INH and ETH, or the necessary enzymes are dysfunctional (13). NITD-916, on the other hand, targets InhA directly and eliminates the need for prodrug conversion (14). NITD-916 is a 4-hydroxy-2-pyridone previously identified in a phenotypic screen of *Mycobacterium tuberculosis* that effectively inhibits InhA and MA synthesis (15). NITD-916 exhibited activity toward INH-resistant *M. tuberculosis in vitro* and *in vivo* in previous studies, indicating its potential use clinically (15, 16). Indeed, the InhA pathway offers an important target for the development of novel anti-*M*. *abscessus* therapies.

Currently, no compounds that target InhA have received FDA approval to treat *M. abscessus* infections (13, 17–19). Alcaraz et al. demonstrated the activity of NITD-916 against *M. abscessus* growing within macrophages, as well as organoids, *in vitro* (17). The evidence validates the InhA pathway as an excellent target for further exploration in anti-*M*. *abscessus* drug development programs (19). Further experiments are required to evaluate the efficacy of NITD-916 *in vivo* and its compatibility with other drugs used clinically. The goal of the study described herein was to assess the activity of NITD-916 against a large collection of clinical *M. abscessus* isolates growing *in vitro*. The compatibility of NITD-916 with other drugs and its effect on the growth of *M. abscessus* in a mouse model of pulmonary lung infection are also presented. Importantly, the combination of *in vitro* and *in vivo* NITD-916 data underscores the potential for clinical use of this compound and paves the way for evaluating other direct InhA inhibitors. Targeting the InhA pathway may offer valuable translational interest in developing new therapeutic regimens to treat *M. abscessus* infections.

## RESULTS

### Comparison with clinically available drugs identifies NITD-916 as a potent inhibitor of *M. abscessus*

A study was undertaken to compare the antimicrobial activity of NITD-916 with the activities of 14 clinically available antibiotics to treat *M. abscessus* infections; the activities of NITD-916, as well as the other drugs, were evaluated. NITD-916 exhibited greater antibacterial activity toward the *M. abscessus* reference strains than most drugs used clinically (Table 1). In contrast, INH and ETH were poorly active against *M. abscessus* despite also targeting the MA synthetic pathway. Moreover, the minimal inhibitory concentrations (MICs) of NITD-916 were lower than most other anti-NTM antibiotics with different mechanisms of action. The susceptibility of 10 other NTM reference strains was also tested. NITD-916 was active against *Mycobacterium fortuitum, Mycobacterium peregrinum, Mycobacterium avium*, and *Mycobacterium intracellulare* reference strains (Table S1).

**TABLE 1:** Antibiotic activity for two *M. abscessus* reference strains

Antibiotic class	Target	Antibiotic	MIC for ATCC 19977 (mg/L)	MIC for CIP 108297 (mg/L)
Pyridines	InhA	NITD-916	0.125	0.25
		Isoniazid	>128^*a*^	>128^*a*^
		Ethionamide	32	32
Macrolides	50S ribosomal subunit	Clarithromycin	0.5	1
Aminoglycosides	30S ribosomal subunit	Amikacin	32	64
β-lactams	Peptidoglycan biosynthesis	Imipenem	32	32
		Cefoxitin	16	32
Fluoroquinolones	DNA gyrase	Moxifloxacin	2	4
		Ciprofloxacin	8	16
Rifamycins	RNA polymerase	Rifabutin	4	8
Oxazolidinones	50S ribosomal subunit	Linezolid	8	8
Glycylcyclines	30S ribosomal subunit	Tigecycline	0.5	2
Tetracycline	30S ribosomal subunit	Omadacycline	4	8
Diarylquinolines	ATP synthetase	Bedaquiline	0.125	0.25
Riminophenazines	NADH-quinone oxidoreductase	Clofazimine	0.5	2

^*a*^ >128 indicates a MIC value greater than the highest concentration tested.

### NITD-916 is active *in vitro* against clinical *M. abscessus* isolates

**Fig 1:**
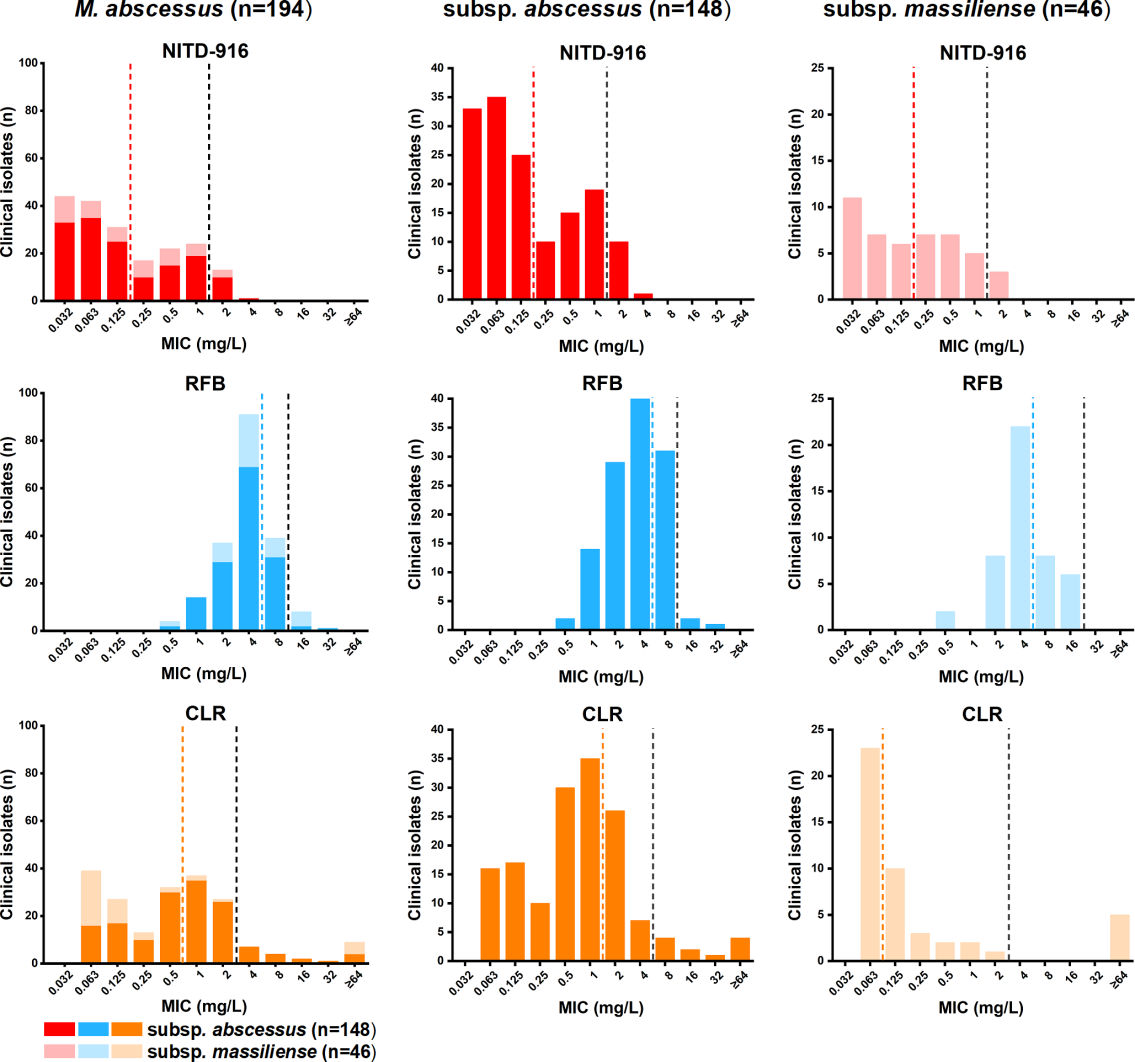
Distribution of the MICs of NITD-916, RFB, and CLR for 194 clinical *M. abscessus* isolates. The *M. abscessus* isolates are comprised of the two subspecies: subsp. *abscessus* (*n* = 148) and subsp. *massiliense* (*n* = 46). MIC_50_ and MIC_90_ in the bar charts are indicated by colored dotted and black dashed vertical lines, respectively. MIC values were determined according to the CLSI document M24, third edition, for aerobic bacteria (20).

The activity of NITD-916 was subsequently tested against 194 clinical *M. abscessus* isolates (148 subsp. *abscessus* and 46 subsp. *massiliense*) (Fig. 1). NITD-916 exhibited comparable anti-*M*. *abscessus* activity toward all *M. abscessus* growing *in vitro*; the MICs ranged from 0.032 to 4 mg/L. The MIC_50_ and MIC_90_ for all isolates tested were 0.125 and 1 mg/L, respectively. The MIC_50_ and MIC_90_ for subsp. *abscessus* and subsp. *massiliense* were the same. Furthermore, the MICs of NITD-916 for clinical *M. abscessus* isolates were significantly lower than either clarithromycin (CLR) or rifabutin (RFB) (Wilcoxon signed rank test, *P* < 0.01; Table S2). The MICs of CLR ranged from 0.063 to ≥64 mg/L; the MIC_50_ and MIC_90_ were 1 and 4 mg/L, respectively. The MICs of RFB ranged from 0.5 to 32 mg/L; the MIC_50_ and MIC_90_ were 4 and 8 mg/L, respectively.

The detailed MIC distribution of all smooth (S) and rough (R) clinical isolates is shown in Table S2. In general, the MICs for the S isolates were twofold to fourfold lower than for the R isolates. Variation among isolates was apparent (Wilcoxon signed rank test, *P* < 0.01, Table S2; Fig. S2). Notably, the MICs of NITD-916 were lower for S and R isolates growing in Middlebrook 7H9 broth than in Cation-Adjusted Mueller-Hinton Broth (Table S3). This difference disappeared, however, when the medium was enriched with acid-albumin-dextrose-catalase (OADC). The S and R isolates were equally susceptible to NITD-916 cultured at either 30°C or 37°C (Table S4).

### NITD-916 does not antagonize clinically available anti-*M*. *abscessus* drugs

Two *M. abscessus* reference strains and eight clinical isolates were selected at random to investigate the interaction between NITD-916 and 10 clinically available anti-*M*. *abscessus* drugs (Table 2). NITD-916 synergized (fractional inhibitory concentration index [FICI] ≤ 0.5) with CLR, inhibiting the growth of 2 out of 5 subsp. *massiliense* isolates, while the interaction for all subsp. *abscessus* isolates was indifferent. No antagonism between NITD-916 and any of the drugs was observed; indifference (FICI = 0.75 to 2) was the most commonly observed interaction.

**TABLE 2:** FICI of NITD-916 combined with agents clinically available to treat *M. abscessus* infections

		FICI (NITD-916 + agent indicated)^*a*^									
Isolate or reference strain	Subspecies	CLR^***b***^	AMK	IPM	FOX	MXF	RFB	LZD	CFZ	TGC	BDQ
ATCC 19977	*abscessus*	1.25	0.75	0.75	1	1.5	0.75	0.75	1	1.5	2
CIP 108297	*massiliense*	0.5	1	1.25	1	1	1.5	1.5	1	1.5	1.25
G169	*abscessus*	1.5	2	1	1.5	1.25	2	1.5	1	2	1
G95	*massiliense*	0.5	2	1.5	2	1.5	1	1.5	1	1.25	2
G120	*abscessus*	1	1.5	1.5	0.75	1.25	0.75	1.25	1.5	0.75	1.5
A205	*massiliense*	1.5	1	1.5	1	1.5	1.5	1	1	1.25	1.5
G201	*abscessus*	1.5	1.5	1	1	1.5	2	1	1.5	1.5	1
G188	*massiliense*	1.5	0.75	2	1.5	2	1.5	1.5	1.5	2	1
A249	*abscessus*	2	1	1.5	2	1	1.5	0.75	1.5	1.5	1.5
G220	*massiliense*	1	1.5	2	1.5	1	1.5	1.5	1	1.5	1

^*a*^ FICI (fractional inhibitory concentration index) = ([MIC of NITD-916 in combination/MIC of NITD-916 alone] + [MIC of the second antibiotic in combination/MIC of the second antibiotic alone]). Synergy, FICI ≤ 0.5; indifference, FICI > 0.5 and ≤4; antagonism, FICI > 4.

^*b*^ CLR, clarithromycin; AMK, amikacin; IPM, imipenem; FOX, cefoxitin; MXF, moxifloxacin; RFB, rifabutin; LZD, linezolid; CFZ, clofazimine; TGC, tigecycline; BDQ, bedaquiline.

### NITD-916 is bacteriostatic for *M. abscessus*

**Fig 2:**
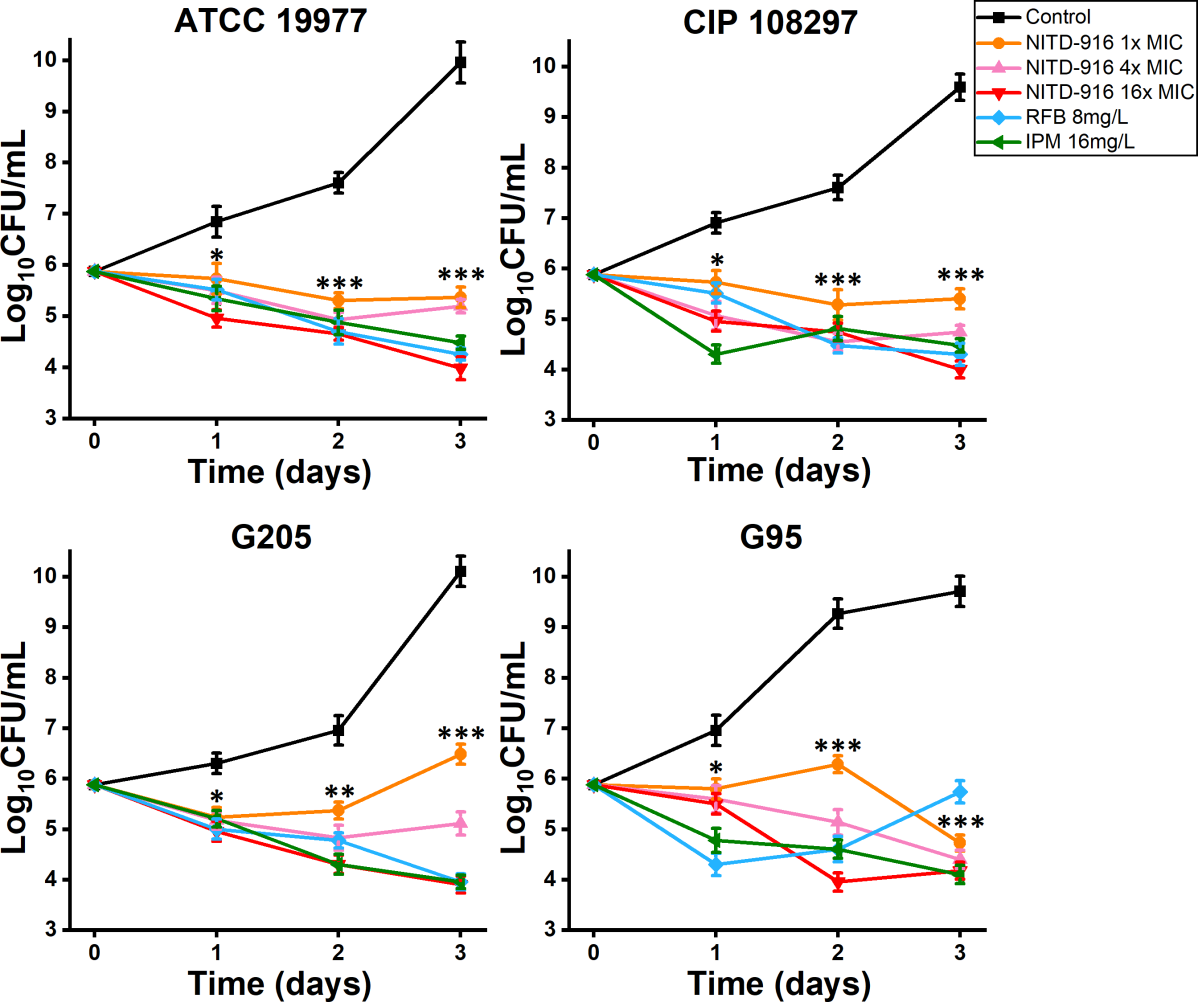
Relative extracellular antimicrobial activities of NITD-916, RFB, and IPM. The number of reference strains ATCC 19977, subsp. *abscessus* and CIP 108297, subsp. *massiliense*; and clinical isolates G205, subsp. *abscessus* and G95, subsp. *massiliense* incubated with or without NITD-916, RFB, or IPM was quantified at the times indicated. Data are the means ± standard deviation of three replicates. Drug-free control is significantly greater than the lowest concentration of NITD-916 (1× MIC): *, *P* < 0.05; **, *P* < 0.01; ***, *P* < 0.001 (Mann-Whitney *U*-test).

The minimum bactericidal concentration (MBC) and MIC values of NITD-916 for *M. abscessus* were assessed using ATCC 19977, CIP 108297, and eight randomly selected isolates. The MICs ranged from 0.063 to 1 mg/L (Table S5). Higher concentrations were required to kill the isolates tested; the MBC values ranged from 0.5 to 32 mg/L. The MBC/MIC ratios ranged from 8 to 64, indicating a bacteriostatic effect. To evaluate further the activity of NITD-916, time-kill kinetics assays were performed on reference strains ATCC 19977, CIP 108297, and clinical isolates G205, G95. Compared to the drug-free control, significant reductions were observed in the growth of the four reference strains and clinical isolates treated with all NITD-916 concentrations tested (Fig. 2). Treatment with 16× MIC NITD-916 resulted in a greater decrease in *M. abscessus* colony-forming units (CFU) compared to the decrease observed in cultures treated with 8 mg/L RFB or 16 mg/L imipenem (IPM).

### NITD-916 inhibits the intracellular growth of *M. abscessus*

**Fig 3:**
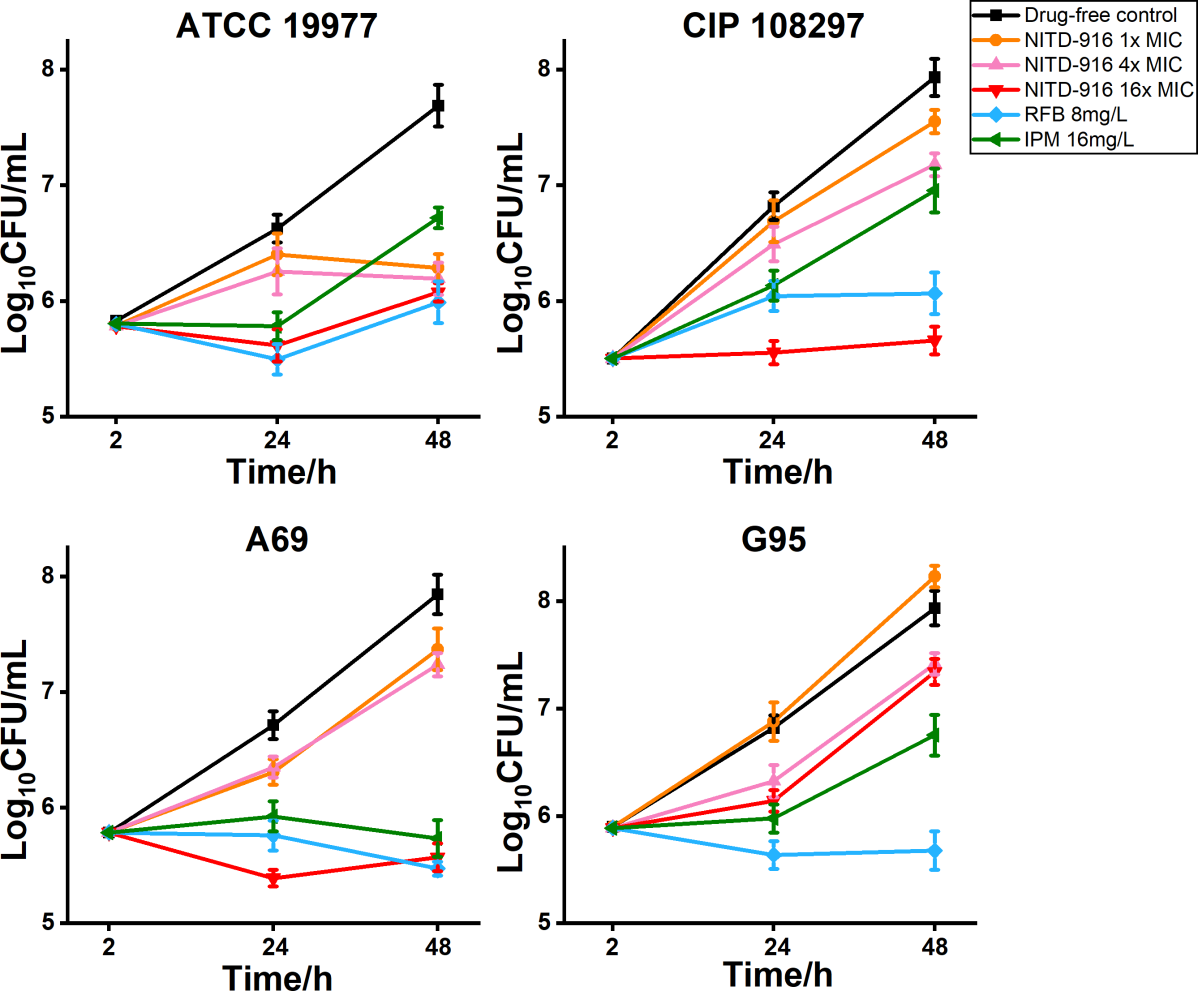
Relative intracellular antimicrobial activities of NITD-916, RFB, and IPM. The number of reference strain ATCC 19977, subsp. *abscessus*; reference strain CIP 108297, subsp. *massiliense*; and clinical isolate A69, subsp. *abscessus*; and clinical isolate G95, subsp. *massiliense* surviving within macrophages was quantified at the times indicated. Data are the means ± standard deviation of three replicates. Numbers of organisms treated with 16× MIC NITD-916, 8 mg/L RFB or 16 mg/L IPM at 24 and 48 hours are significantly less than the drug-free control: *P* < 0.01 (Mann-Whitney *U*-test).

Even the lowest concentrations of NITD-916, i.e., 1× MIC and 4× MIC, inhibited the intracellular growth of *M. abscessus*, reference strains ATCC 19977 and CIP 108297, and clinical isolates A69 and G95 (Fig. 3). NITD-916 (16× MIC) significantly decreased the number of intracellular mycobacteria present at 24 and 48 hours. The intracellular CFUs of reference strain ATCC 19977 and clinical isolates A69 and G95 were reduced by 2.1 ± 0.5 log_10_ CFUs at 48 hours relative to the drug-free control. This reduction was similar to 8 mg/L RFB, but greater than that found for 16 mg/L IPM-treated macrophages. NITD-916 (16× MIC) also significantly reduced the counts of reference strain CIP 108297 by 2.3 log_10_ CFUs at 48 hours. By comparison, 8 mg/L RFB and 16 mg/L IPM reduced intracellular CIP 108297 by 1.9 log_10_ and 1.0 log_10_ CFUs, respectively. Consequently, the antimycobacterial activity of NITD-916 increased with increasing drug concentrations and was comparable to or greater than that exhibited by RFB and IPM.

### NITD-916 inhibits the replication of *M. abscessus* in a mouse model of lung infection

**Fig 4:**
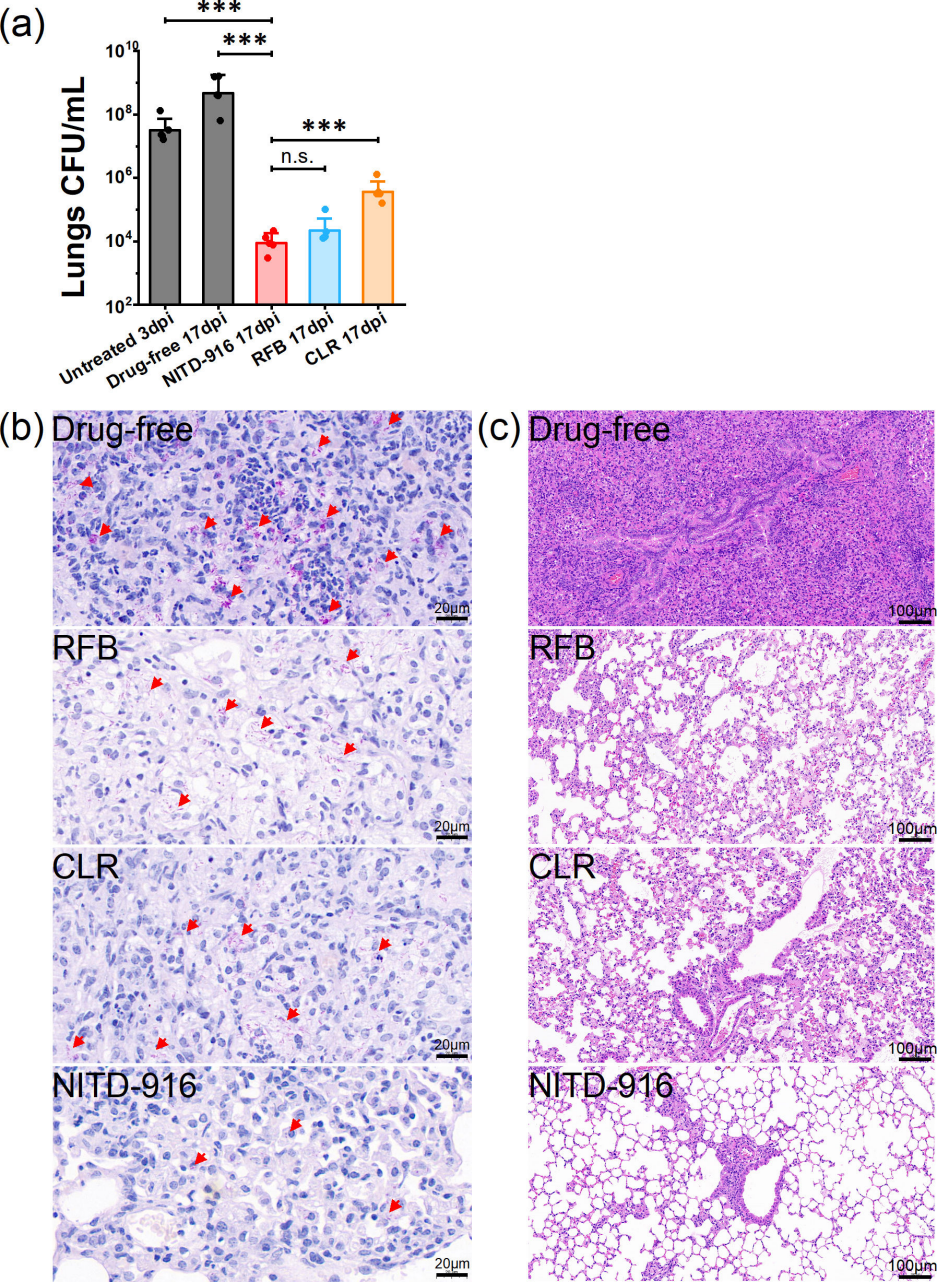
NITD-916 exhibits anti-*M*. *abscessus* activity in mice. (**a**) CFU assay. Data are the mean CFUs ± standard deviation determined at 3 days (untreated) or 17 days post-infection, i.e., 14 days post-administration of 100 mg/kg NITD-916, 100 mg/kg RFB, or 200 mg/kg CLR, *n* = 5. Statistically different: n*.*s*.*, no significance; ***, *P* < 0.001 (Mann-Whitney *U*-test). The lungs were dissected, processed, sectioned, and stained. (**b**) Acid-fast stained *M. abscessus* in mouse lung tissue; acid-fast bacilli are stained magenta. The scale bar represents 20 µm. (**c**) Histopathologic assessment of mouse lungs stained with hematoxylin and eosin (H&E). The scale bar represents 100 µm.

Experiments were conducted to evaluate and compare the effects of NITD-916, RFB, and CLR on the growth of *M. abscessus in vivo*. NITD-916 treatment resulted in a significant (5.6 log_10_ CFUs) reduction in the bacterial load in the lungs of mice compared to the untreated, drug-free control group (*P* < 0.001) (Fig. 4a). In contrast, CLR treatment reduced the bacterial load in the lungs of mice <3 log_10_ CFUs relative to the control animals; RFB exhibited greater efficacy than CLR, comparable to NITD-916. Treatment with 100 mg/kg NITD-916 significantly reduced the *M. abscessus* load evident in acid-fast stained tissue sections (Fig. 4b). Treatment with CLR or RFB also reduced the number of acid-fast stained bacilli found in tissue sections, but not to the same extent as found in sections derived from the NITD-916-treated animals.

Hematoxylin and eosin (H&E) stained tissue sections revealed consolidated lung tissue, including severe alveolar wall thickening, inflammatory cell infiltration, and diapedesis of the erythrocytes in the lungs of the drug-free group of mice on day 17 post-infection (Fig. 4c). In marked contrast, lung lesions were negligible and pathological changes were rare in the NITD-916-treated group. H&E staining also revealed less tissue damage in the lungs of *M. abscessus*-infected mice treated with CLR or RFB, though the damage was more severe than that observed in the lungs of the NITD-916-treated mice.

## DISCUSSION

*M. abscessus* has been called “a new antibiotic nightmare” due to its resistance to most antibiotics (5). Conceivably, the findings in this study will facilitate the urgent need to discover a new antimicrobial agent to treat *M. abscessus* infections.

NITD-916 was active against a large panel of 194 clinical isolates (MIC ≤ 4 mg/L) and was equally effective in treating *M. abscessus* subsp. *abscessus* and subsp. *massiliense*. NITD-916 suppressed replication in a dose-dependent manner in an intracellular killing assay conducted *in vitro*; bacterial growth was reduced by 0.5–2.6 log_10_ CFUs compared to untreated control macrophages. NITD-916 possessed anti-*M*. *abscessus* activity that was comparable to that displayed by RFB and IPM. The basis for higher MICs of NITD-916 for R, compared to S isolates, is unclear. Conceivably, the absence of glycopeptidelipids (GPL), which leads to enhanced cell wall hydrophobicity and bacterial aggregation typical of R morphotypes, prevents NITD-916 binding to cell wall targets (21, 22). MA, the major hydrophobic component of the mycomembrane, might replace GPL, thereby increasing the amount and exposure of MA present in the cell wall of R morphotypes. Whether these differences relate to the effects of NITD-916 on MA biosynthesis remains to be determined.

There are several reports regarding the anti-*M*. *abscessus* activity of InhA inhibitors, most of which belong to a new class of inhibitors: 4-hydroxy-2-pyridones (including NITD-529, NITD-564), triclosan, and pyridomycin. Triclosan, a type of diphenyl ether, is generally considered a promising compound that directly inhibits InhA and is active against *M. abscessus*, exhibiting a MIC of 6.73 mg/L *in vitro* (18). Several drawbacks, however, limit the use of triclosan as an anti-*M*. *abscessus* agent: suboptimal bioavailability and limited aqueous solubility (23). Pyridomycin, a natural product that inhibits InhA, suppresses *M. abscessus* growth *in vitro* (MIC = 3.58 mg/L) (24); its effect on growth *in vivo* has not been reported. NITD-529 and NITD-564 were initially developed to treat *M. tuberculosis* infections; their MICs for *M. tuberculosis* were higher than NITD-916. Consequently, the data obtained for NITD-529 and NITD-564 in a previous study only suggest their potential anti-*M*. *abscessus* activity (15).

Though NITD-916 appeared to be more active against *M. abscessus* than either CLR or RFB, it exhibited a narrower spectrum of anti-NTM activity. NITD-916 expressed greater antibacterial activity toward *M. fortuitum* and *M. peregrinum* reference strains than CLR and RFB. Its activity toward *M. avium* and *M. intracellulare* was also significant. However, its activity against *Mycobacterium kansasii*, *Mycobacterium scrofulaceum*, *Mycobacterium szulgai,* and *Mycobacterium xenopi* was comparatively weaker than either CLR or RFB. Interestingly, both INH and ETH were active against *M. kansasii*, possibly correlating with their pre-activation (25).

Effective regimens used to treat *M. abscessus* infections require a combination of antimicrobials; macrolides are essential (7, 26). Macrolides alone, however, are insufficient to eradicate *M. abscessus* infections in profoundly ill patients (6, 27). In this regard, NITD-916 exhibited 20% (2/10) synergy and 80% (8/10) indifference when combined with CLR in the study reported here. Synergy between NITD-916 and CLR inhibited the growth of certain *M. abscessus* subsp. *massiliense* isolates, but not subsp. *abscessus* isolates. The gene *erm*(41) confers inducible macrolide resistance to subsp. *abscessus*, but its absence in subsp. *massiliense* suggests a plausible explanation for the limited synergy between NITD-916 and CLR (28). Subsp. *massiliense* was chosen for infection of the mouse model because its susceptibility to macrolides enabled the use of CLR as a positive control in accordance with previous studies (29, 30). NITD-916 did not antagonize any of the anti-*M*. *abscessus* drugs used most frequently. Therefore, NITD-916 is compatible and could readily be incorporated into the current anti-*M*. *abscessus* treatment approaches.

The efficacy of NITD-916 and RFB was comparable to and greater than CLR in the mouse model described herein. Notably, histopathological improvements were more significant in the NITD-916-treated group than in either the RFB- or CLR-treated group. This is likely due to the inhibitory effects of NITD-916 on inflammatory signaling pathways that contribute to lung damage. NITD-916 exhibits good oral bioavailability (66% at 25 mg/kg) with a peak serum concentration of 39.23 µM *in vivo*; however, its distribution to mice lungs is inefficient (15). Related analogs, e.g., replacing the R3 phenyl with an alternative ring system, could be synthesized to address the challenges of low solubility and lipophilicity (15). Such a change could improve volume distribution, reduce plasma protein binding, and enhance bioavailability. A phosphate ester prodrug, NITD-113, is rapidly hydrolyzed by intestinal alkaline phosphatases and readily converted into NITD-916 in mice, leading to improved pulmonary distribution (15, 19). Further investigations should focus on inhalation formulations that optimize lung retention time, mucosal penetration efficiency, and local conversion rates. While prodrug strategies may mitigate certain NITD-916 limitations, essential parameters that include conversion stability, metabolic pathway, formulation safety, and dosage require rigorous evaluation.

Sequencing the *inhA_MAB_* locus, which encodes the enzyme enoyl-ACP reductase InhA_MAB_ (MAB_2722c), revealed the presence of single-nucleotide polymorphisms (SNPs) across 194 clinical isolates (Table S2). These SNPs resulted in amino acid changes at positions 48, 58, 72, 82, 84, and 85, but did not include the Gly96 mutations previously associated with *InhA_MAB_* inducible resistance (17). Despite these mutations, the MICs for the isolates remained between 0.032 and 2 mg/L, indicating no intrinsic resistance of *M. abscessus* relating to *inhA_MAB_*. Interestingly, one subsp. *abscessus* R isolate (G200) exhibiting the highest MIC value (4 mg/L) among all isolates possessed mutations in the efflux pump MAB_1409 (tap-like) rather than in *inhA_MAB_*, suggesting a possible alternate resistance mechanism. Reportedly, the major facilitator superfamily efflux pump gene *Mab_1409* (*tap-like*) mediates drug efflux and CLR resistance (31, 32). The MICs of NITD-916 for 38 clinical isolates with missense mutations in *Mab_1409* were higher than the MIC for wild-type ATCC 19977 (Mann-Whitney *U*-test, *P* < 0.05) (Table S2). We hypothesize that missense mutations in *Mab_1409* might upregulate the function of MAB_1409 efflux pumps and increase the resistance to NITD-916.

This study has several limitations. First, the effects of NITD-916 on all clinical isolates were compared with the effects of CLR and RFB, neither of which targets InhA. The poor activity exhibited by INH toward *M. abscessus* negates comparison in the present study. Secondly, only *M. abscessus* subsp. *abscessus* and subsp. *massiliense* isolates were included in this study. Subsp. *bolletii* was not investigated because none of the 194 clinical isolates used and characterized in previous studies belonged to subsp. *bolletii* (29, 33).

In conclusion, the discovery of InhA as the molecular target of anti-*M*. *abscessus* therapy emphasizes that inhibition of InhA is an attractive strategy to treat *M. abscessus* infections. NITD-916 was highly effective in inhibiting *M. abscessus* growth both *in vitro* and *in vivo* and was compatible with antibiotics frequently used to treat *M. abscessus* infections. As such, NITD-916 represents an advanced lead compound for incorporation into novel therapeutic anti-*M*. *abscessus* regimens.

## MATERIALS AND METHODS

### Drugs and compounds

Isoniazid, ethionamide, clarithromycin, amikacin, imipenem, cefoxitin, moxifloxacin, ciprofloxacin, linezolid, tigecycline, omadacycline, bedaquiline, and clofazimine were purchased from MedChemExpress (New Jersey, USA). Rifabutin and NITD-916 were purchased from Sigma-Aldrich (Darmstadt, Germany) and TargetMol Chemicals Inc. (Shanghai, China), respectively.

### Strains and culture media

Twelve NTM reference strains enumerated in Table S1 were obtained from the Type Culture Collection (Manassas, VA, USA) and the Biological Resource Center of Institute Pasteur (Paris, France) as described previously (27). Clinical isolates used in this study were obtained from Shanghai Pulmonary Hospital as described previously (27). All strains were grown at 37°C with agitation in Middlebrook 7H9 medium supplemented with 10% OADC (BD Sciences, New Jersey, USA) and 0.05% Tween 80 or on Middlebrook 7H10 agar (BD Difco, New Jersey, USA) supplemented with 10% OADC (7H10_OADC_). All *M. abscessus* isolates were sequenced; the full genome sequence of each isolate was published and is available at DDBJ/ENA/GenBank (BioProject PRJNA488058, PRJNA448987, and PRJNA398137) (28, 33).

### Antimicrobial susceptibility testing

MICs of NITD-916 and other drugs against NTM reference strains and clinical isolates were determined by the broth microdilution method in accordance with Clinical and Laboratory Standards Institute (CLSI) document M24, third edition (20). *M. abscessus* subsp. *abscessus* ATCC 19977, subsp. *massiliense* CIP 108297, and eight randomly selected clinical *M. abscessus* isolates were used in assays to determine MBC. To determine bactericidal activity, cultures were grown as described for the MIC determinations, except that the cultures were plated on agar after 3–5 days of incubation. Each well was re-suspended manually, and 10 µL of the cultures from the first clear well and those thereafter were plated at different dilutions on 7H10_OADC_ agar plates. The plates were incubated at 37°C for an additional 3–5 days, and the colonies were counted. MBC was defined as the minimum drug concentration that prevented 99.9% bacterial growth expressed as CFUs/mL. An antibiotic was considered bactericidal if the MBC/MIC ratio was <4 or bacteriostatic if the ratio was >4 (34).

### Time-kill kinetics assay

The potency of the killing kinetics of NITD-916 was evaluated using *M. abscessus* subsp. *abscessus* ATCC 19977, subsp. *massiliense* CIP 108297, clinical subsp. *abscessus* isolate G205, and clinical subsp. *massiliense* isolate G95. Tubes of 7H9_OADC_ broth containing NITD-916 at 1× MIC, 4× MIC, or 16× MIC, and 8 mg/L RFB or 16 mg/L IPM were inoculated with 5 × 10^5^ CFU/mL of one of the reference strains or clinical isolates growing exponentially. The tubes were incubated for 3 days at 37°C. The CFUs were quantified by plating serial dilutions of bacteria on 7H10_OADC_ agar plates after 0, 1, 2, and 3 days of incubation.

### Drug synergy

Synergy between NITD-916 and clarithromycin, amikacin, imipenem, cefoxitin, moxifloxacin, rifabutin, linezolid, clofazimine, tigecycline, or bedaquiline was assessed *in vitro* using the broth microdilution checkerboard titration assay (35). Two *M. abscessus* reference strains and eight clinical isolates were used. Synergy test results were interpreted based upon the FICI, which was calculated using the following formula: FICI = (MIC of antibiotic A in combination/MIC of antibiotic A alone) + (MIC of NITD-916 in combination/MIC of NITD-916 alone). Drug interactions were classified as synergistic (FICI ≤ 0.5), indifferent (0.5 < FICI ≤ 4), or antagonistic (FICI > 4).

### Intracellular antibacterial activity assay

Macrophages derived from the human THP-1 monocyte cell line (purchased from Cell Bank/Stem Cell Bank, Chinese Academy of Sciences) were infected at a multiplicity of infection = 10 with *M. abscessus* reference strains ATCC 19977 and CIP 108297, and clinical isolates subsp. *abscessus* A69 and subsp*. massiliense* G95 as described previously (27). RPMI medium (1 mL) supplemented with 10% fetal bovine serum (Sigma-Aldrich, Darmstadt, Germany) containing DMSO, 8 mg/L RFB, 16 mg/L IPM, or 1× MIC, 4× MIC, 16× MIC NITD-916 was then added. The cells were lysed with 0.05% Triton X-100 after 2, 24, or 48 hours of infection, and the CFUs were quantified by plating serial dilutions of lysates on 7H10_OADC_ agar plates.

### Mouse model of *M. abscessus* infection

All animal experiments were approved by the Institutional Animal Ethics Committee of Shanghai Pulmonary Hospital (Protocol No. K24-309). To establish a neutropenic mouse model, 6-week-old male BALB/c mice (18 to 20 g) were injected intraperitoneally with 150 mg/kg cyclophosphamide on days −4 and −1 prior to infection (day 0). The mice were then infected intranasally with 1 × 10^6^ CFU *M*. *abscessus* CIP 108297. On day 3 post-infection, five mice were euthanized, and the CFUs in the lungs were quantified to assess the efficacy of the model. The remaining 20 mice were randomly divided into four treatment groups (*n* = 5/group): group 1: control, administered MicroEmulsion Pre-Concentrate (MEPC) intragastrically daily for 14 days); group 2: 100 mg/kg NITD-916 in MEPC administered intragastrically daily for 14 days; group 3: 100 mg/kg RFB in PBS containing 0.5% methylcellulose and 0.1% Tween 80 administered intragastrically daily for 14 days; and group 4: 200 mg/kg CLR in PBS with 0.5% methylcellulose and 0.1% Tween 80 administered intragastrically daily for 14 days. MEPC consists of 10% Capmul MCM, 25% Cremophor-RH40, 15% ethanol, and 50 mM phosphate buffer (15). A 100 mg/kg dose of NITD-916 was selected based upon prior safety and dose-dependent efficacy data reported previously in a study of *M. tuberculosis*-infected mice (15); CLR dosing followed established protocols (27, 36).

On day 17 post-infection, the mice were euthanized. The upper lobe of the right lung was dissected, fixed for 24 hours in 4% paraformaldehyde, and sectioned for histopathology. Tissue sections were subjected to H&E and acid-fast staining. The left lungs were homogenized, serially diluted 10-fold, and plated on 7H10_OADC_ agar. The bacterial burden was determined from the number of colonies that grew after 5 days of incubation at 37°C.

### Statistical evaluation

Statistical differences between study groups were determined using the Mann-Whitney *U*-test or Student’s *t*-test; *P* < 0.05 was considered statistically significant. Computations were performed using OriginPro 2024 (OriginLab, USA). Genomic sequence mutations were determined using SnapGene v7.0.2 (GSL Biotech, USA).

## SUPPLEMENTAL MATERIAL

Supplemental material aac.00249-25-s0001.pdf Tables S1 to S4; Fig. S1.

ASM does not own the copyrights to Supplemental Material that may be linked to, or accessed through, an article. The authors have granted ASM a non-exclusive, world-wide license to publish the Supplemental Material files. Please contact the corresponding author directly for reuse.

## References

[1] Johansen MD, Herrmann JL, Kremer L. 2020. Non-tuberculous mycobacteria and the rise of Mycobacterium abscessus. Nat Rev Microbiol 18:392–407. doi:10.1038/s41579-020-0331-1 32086501

[2] Bernut A, Dupont C, Ogryzko NV, Neyret A, Herrmann JL, Floto RA, Renshaw SA, Kremer L. 2019. CFTR protects against Mycobacterium abscessus infection by fine-tuning host oxidative defenses. Cell Rep 26:1828–1840. doi:10.1016/j.celrep.2019.01.071 30759393 PMC7618368

[3] Jarand J, Levin A, Zhang L, Huitt G, Mitchell JD, Daley CL. 2011. Clinical and microbiologic outcomes in patients receiving treatment for Mycobacterium abscessus pulmonary disease. Clin Infect Dis 52:565–571. doi:10.1093/cid/ciq237 21292659

[4] Marty PK, Yetmar ZA, Gerberi DJ, Escalante P, Pennington KM, Mahmood M. 2023. Risk factors and outcomes of non-tuberculous mycobacteria infection in lung transplant recipients: a systematic review and meta-analysis. J Heart Lung Transplant 42:264–274. doi:10.1016/j.healun.2022.10.004 36334962

[5] Nessar R, Cambau E, Reyrat JM, Murray A, Gicquel B. 2012. Mycobacterium abscessus: a new antibiotic nightmare. J Antimicrob Chemother 67:810–818. doi:10.1093/jac/dkr578 22290346

[6] Daley CL, Iaccarino JM, Lange C, Cambau E, Wallace RJ, Andrejak C, Böttger EC, Brozek J, Griffith DE, Guglielmetti L, Huitt GA, Knight SL, Leitman P, Marras TK, Olivier KN, Santin M, Stout JE, Tortoli E, van Ingen J, Wagner D, Winthrop KL. 2020. Treatment of nontuberculous mycobacterial pulmonary disease: an official ATS/ERS/ESCMID/IDSA clinical practice guideline. Eur Respir J 56:2000535. doi:10.1183/13993003.00535-2020 32636299 PMC8375621

[7] Kwak N, Dalcolmo MP, Daley CL, Eather G, Gayoso R, Hasegawa N, Jhun BW, Koh WJ, Namkoong H, Park J, Thomson R, Ingen J, Zweijpfenning SMH, Yim JJ. 2019. Mycobacterium abscessus pulmonary disease: individual patient data meta-analysis. Eur Respir J 54:1801991. doi:10.1183/13993003.01991-2018 30880280

[8] Andréjak C, Thomsen VØ, Johansen IS, Riis A, Benfield TL, Duhaut P, Sørensen HT, Lescure FX, Thomsen RW. 2010. Nontuberculous pulmonary mycobacteriosis in Denmark: incidence and prognostic factors. Am J Respir Crit Care Med 181:514–521. doi:10.1164/rccm.200905-0778OC 20007929

[9] Jhun BW, Moon SM, Jeon K, Kwon OJ, Yoo H, Carriere KC, Huh HJ, Lee NY, Shin SJ, Daley CL, Koh W-J. 2020. Prognostic factors associated with long-term mortality in 1445 patients with nontuberculous mycobacterial pulmonary disease: a 15-year follow-up study. Eur Respir J 55:1900798. doi:10.1183/13993003.00798-2019 31619468

[10] Jankute M, Cox JAG, Harrison J, Besra GS. 2015. Assembly of the mycobacterial cell wall. Annu Rev Microbiol 69:405–423. doi:10.1146/annurev-micro-091014-104121 26488279

[11] Halloum I, Carrère-Kremer S, Blaise M, Viljoen A, Bernut A, Le Moigne V, Vilchèze C, Guérardel Y, Lutfalla G, Herrmann JL, Jacobs WR Jr, Kremer L. 2016. Deletion of a dehydratase important for intracellular growth and cording renders rough Mycobacterium abscessus avirulent. Proc Natl Acad Sci U S A 113:E4228–E4237. doi:10.1073/pnas.1605477113 27385830 PMC4961194

[12] Banerjee A, Dubnau E, Quemard A, Balasubramanian V, Um KS, Wilson T, Collins D, de Lisle G, Jacobs WR Jr. 1994. inhA, a gene encoding a target for isoniazid and ethionamide in Mycobacterium tuberculosis. Science 263:227–230. doi:10.1126/science.8284673 8284673

[13] Gagliardi A, Selchow P, Luthra S, Schäfle D, Schulthess B, Sander P. 2020. KatG as counterselection marker for nontuberculous mycobacteria. Antimicrob Agents Chemother 64:e02508-19. doi:10.1128/AAC.02508-19 32317266 PMC7179591

[14] Encinas L, O’Keefe H, Neu M, Remuiñán MJ, Patel AM, Guardia A, Davie CP, Pérez-Macías N, Yang H, Convery MA, et al.. 2014. Encoded library technology as a source of hits for the discovery and lead optimization of a potent and selective class of bactericidal direct inhibitors of Mycobacterium tuberculosis InhA. J Med Chem 57:1276–1288. doi:10.1021/jm401326j 24450589

[15] Manjunatha UH, S Rao SP, Kondreddi RR, Noble CG, Camacho LR, Tan BH, Ng SH, Ng PS, Ma NL, Lakshminarayana SB, Herve M, Barnes SW, Yu W, Kuhen K, Blasco F, Beer D, Walker JR, Tonge PJ, Glynne R, Smith PW, Diagana TT. 2015. Direct inhibitors of InhA are active against Mycobacterium tuberculosis. Sci Transl Med 7:269ra3. doi:10.1126/scitranslmed.3010597 PMC4383039 25568071

[16] Ng PS, Manjunatha UH, Rao SPS, Camacho LR, Ma NL, Herve M, Noble CG, Goh A, Peukert S, Diagana TT, Smith PW, Kondreddi RR. 2015. Structure activity relationships of 4-hydroxy-2-pyridones: a novel class of antituberculosis agents. Eur J Med Chem 106:144–156. doi:10.1016/j.ejmech.2015.10.008 26544629

[17] Alcaraz M, Roquet-Banères F, Leon-Icaza SA, Abendroth J, Boudehen YM, Cougoule C, Edwards TE, Kremer L. 2022. Efficacy and mode of action of a direct inhibitor of Mycobacterium abscessus InhA. ACS Infect Dis 8:2171–2186. doi:10.1021/acsinfecdis.2c00314 36107992

[18] Rodriguez R, Campbell-Kruger N, Gonzalez Camba J, Berude J, Fetterman R, Stanley S. 2023. MarR-dependent transcriptional regulation of mmpSL5 induces ethionamide resistance in Mycobacterium abscessus. Antimicrob Agents Chemother 67:e0135022. doi:10.1128/aac.01350-22 36988462 PMC10112066

[19] Alcaraz M, Edwards TE, Kremer L. 2023. New therapeutic strategies for Mycobacterium abscessus pulmonary diseases - untapping the mycolic acid pathway. Expert Rev Anti Infect Ther 21:813–829. doi:10.1080/14787210.2023.2224563 37314394 PMC10529309

[20] Clinical and Laboratory Standards Institute. 2018. CLSI standard document M24. Susceptibility testing of mycobacteria, Nocardia spp., and other aerobic actinomycetes. 3rd ed. Clinical and Laboratory Standards Institute, Wayne, PA. https://clsi.org/standards/products/microbiology/documents/m24/.

[21] Nessar R, Reyrat JM, Davidson LB, Byrd TF. 2011. Deletion of the mmpL4b gene in the Mycobacterium abscessus glycopeptidolipid biosynthetic pathway results in loss of surface colonization capability, but enhanced ability to replicate in human macrophages and stimulate their innate immune response. Microbiology (Reading) 157:1187–1195. doi:10.1099/mic.0.046557-0 21292749

[22] Viljoen A, Viela F, Kremer L, Dufrêne YF. 2020. Fast chemical force microscopy demonstrates that glycopeptidolipids define nanodomains of varying hydrophobicity on mycobacteria. Nanoscale Horiz 5:944–953. doi:10.1039/c9nh00736a 32314749

[23] Vosátka R, Krátký M, Vinšová J. 2018. Triclosan and its derivatives as antimycobacterial active agents. Eur J Pharm Sci 114:318–331. doi:10.1016/j.ejps.2017.12.013 29277667

[24] Hartkoorn RC, Pojer F, Read JA, Gingell H, Neres J, Horlacher OP, Altmann KH, Cole ST. 2014. Pyridomycin bridges the NADH- and substrate-binding pockets of the enoyl reductase InhA. Nat Chem Biol 10:96–98. doi:10.1038/nchembio.1405 24292073

[25] Bakuła Z, Modrzejewska M, Pennings L, Proboszcz M, Safianowska A, Bielecki J, van Ingen J, Jagielski T. 2018. Drug susceptibility profiling and genetic determinants of drug resistance in Mycobacterium kansasii. Antimicrob Agents Chemother 62:e01788-17. doi:10.1128/AAC.01788-17 29437627 PMC5913922

[26] Chen J, Zhao L, Mao Y, Ye M, Guo Q, Zhang Y, Xu L, Zhang Z, Li B, Chu H. 2019. Clinical efficacy and adverse effects of antibiotics used to treat Mycobacterium abscessus pulmonary disease. Front Microbiol 10:1977. doi:10.3389/fmicb.2019.01977 31507579 PMC6716072

[27] Griffith DE. 2019. Mycobacterium abscessus and antibiotic resistance: same as it ever was. Clin Infect Dis 69:1687–1689. doi:10.1093/cid/ciz071 30689764

[28] Nash KA, Brown-Elliott BA, Wallace RJ. 2009. A novel gene, erm(41), confers inducible macrolide resistance to clinical isolates of Mycobacterium abscessus but is absent from Mycobacterium chelonae. Antimicrob Agents Chemother 53:1367–1376. doi:10.1128/AAC.01275-08 19171799 PMC2663066

[29] Wu W, He S, Li A, Guo Q, Tan Z, Liu S, Wang X, Zhang Z, Li B, Chu H. 2022. A novel leucyl-tRNA synthetase inhibitor, MRX-6038, expresses anti-Mycobacterium abscessus activity in vitro and in vivo. Antimicrob Agents Chemother 66:e0060122. doi:10.1128/aac.00601-22 35969055 PMC9487484

[30] Li B, Yang S, Chu H, Zhang Z, Liu W, Luo L, Ma W, Xu X. 2017. Relationship between antibiotic susceptibility and genotype in Mycobacterium abscessus clinical isolates. Front Microbiol 8:1739. doi:10.3389/fmicb.2017.01739 28959242 PMC5603792

[31] Machado D, Coelho TS, Perdigão J, Pereira C, Couto I, Portugal I, Maschmann RDA, Ramos DF, von Groll A, Rossetti MLR, Silva PA, Viveiros M. 2017. Interplay between mutations and efflux in drug resistant clinical isolates of Mycobacterium tuberculosis. Front Microbiol 8:711. doi:10.3389/fmicb.2017.00711 28496433 PMC5406451

[32] Vianna JS, Machado D, Ramis IB, Silva FP, Bierhals DV, Abril MA, von Groll A, Ramos DF, Lourenço MCS, Viveiros M, da Silva PEA. 2019. The contribution of efflux pumps in Mycobacterium abscessus complex resistance to clarithromycin. Antibiotics (Basel) 8:153. doi:10.3390/antibiotics8030153 31540480 PMC6784190

[33] Li B, Ye M, Guo Q, Zhang Z, Yang S, Ma W, Yu F, Chu H. 2018. Determination of MIC distribution and mechanisms of decreased susceptibility to bedaquiline among clinical isolates of Mycobacterium abscessus. Antimicrob Agents Chemother 62:e00175-18. doi:10.1128/AAC.00175-18 29712658 PMC6021634

[34] Maurer FP, Bruderer VL, Ritter C, Castelberg C, Bloemberg GV, Böttger EC. 2014. Lack of antimicrobial bactericidal activity in Mycobacterium abscessus. Antimicrob Agents Chemother 58:3828–3836. doi:10.1128/AAC.02448-14 24752273 PMC4068550

[35] Kaushik A, Ammerman NC, Tasneen R, Story-Roller E, Dooley KE, Dorman SE, Nuermberger EL, Lamichhane G. 2017. In vitro and in vivo activity of biapenem against drug-susceptible and rifampicin-resistant Mycobacterium tuberculosis. J Antimicrob Chemother 72:2320–2325. doi:10.1093/jac/dkx152 28575382 PMC5890701

[36] Kohno Y, Ohno H, Miyazaki Y, Higashiyama Y, Yanagihara K, Hirakata Y, Fukushima K, Kohno S. 2007. In vitro and in vivo activities of novel fluoroquinolones alone and in combination with clarithromycin against clinically isolated Mycobacterium avium complex strains in Japan. Antimicrob Agents Chemother 51:4071–4076. doi:10.1128/AAC.00410-07 17709469 PMC2151420

