# Supplementary material for: An enoyl-ACP reductase inhibitor, NITD-916, expresses anti-Mycobacterium abscessus activity
Source: Antimicrob Agents Chemother. 2025 May 23;69(7):e00249-25. doi: 10.1128/aac.00249-25 (PMC12217450; doi:10.1128/aac.00249-25)
Supplement: Supplemental material — Tables S1 to S4; Fig. S1. [file aac.00249-25-s0001.pdf]

Supplemental Material

TABLE S1. Antimycobacterial spectrum of NITD-916, clarithromycin (CLR) and rifabutin (RFB) for NTM reference strains

	MIC (mg/L)		
**Reference strain**	**NITD-916**	**CLR**	**RFB**
Rapid-growing mycobacteria			
M. abscessus ATCC 19977	0.125	0.5	4
M. massiliense CIP 108297	0.25	1	8
M. smegmatis ATCC 19420	>32[a]	2	>32
M. fortuitum ATCC 6841	0.25	2	16
M. peregrinum ATCC 700686	0.063	4	16
Slow-growing mycobacteria			
M. avium ATCC 25291	0.5	0.5	0.063
M. intracellulare ATCC 13950	0.25	1	0.063
M. kansasii ATCC 12478	16	0.125	0.063
M. gordonae ATCC 14470	>32	0.25	>32
M. scrofulaceum ATCC 19981	>32	0.5	0.063
M. szulgai ATCC 35799	>32	0.25	0.125
M. xenopi ATCC 19250	>32	0.125	0.063

[a]>32 indicates a MIC value greater than the highest concentration tested.

TABLE S2. Effects of *InhA*$_{MAB}$ (MAB_2722c) mutations on the MICs of NITD-916, clarithromycin (CLR) and rifabutin (RFB) for 194 clinical *M. abscessus* isolates

Isolate	Subspecies[a]	Morphology[b]	Nucleotide changes in *inhA*$_{MAB}$	Amino acid changes in InhA$_{MAB}$	Nucleotide changes in *tap-like*	Amino acid changes in MAB_1409c	MIC (mg/L)		
							NITD-916	CLR	RFB
A8	A	S	-	-	-	-	0.032	0.063	0.5
A10	A	R	-	-	-	-	0.063	0.5	4
A25	A	R	-	-	-	-	0.063	0.25	2
A35	A	R	142C>A, 250C>A	E48D, E84D	-	-	0.032	0.5	1
A38	A	S	-	-	-	-	0.5	2	2
A40	A	R	250C>A	E84D	-	-	0.25	4	4
A49	A	R	-	-	166T>C, 782A>G, 1172C>G	I56V, I261T, G391A	0.5	1	2
A51	A	S	-	-	-	-	0.5	1	4
A54	A	S	142C>A	E48D	-	-	0.063	1	1
A58	A	S	-	-	-	-	0.032	0.5	2
A59	A	S	-	-	-	-	0.032	0.5	2
A69	A	S	-	-	-	-	0.032	0.063	4
A73	A	S	-	-	-	-	0.032	0.5	1
A79	A	S	-	-	-	-	0.032	0.25	2
A126	A	S	-	-	-	-	0.125	0.063	2
A137	A	R	-	-	-	-	0.063	1	1
21	A	R	-	-	-	-	0.063	1	1
A176	A	S	142C>A, 250C>A	E48D, E84D	-	-	0.125	0.5	1
A182	A	S	-	-	-	-	0.063	0.25	2
A183	A	S	-	-	-	-	0.032	0.125	2
A189	A	S	-	-	-	-	0.5	0.5	4
A197	A	S	-	-	-	-	0.063	2	1
A213	A	R	-	-	-	-	0.063	0.5	8
A215	A	R	-	-	166T>C, 187G>A, 782A>G, 1172C>G	I56V, L63F, I261T, G391A	1	0.25	4
A217	A	R	-	-	166T>C, 187G>A, 782A>G, 1172C>G	I56V, L63F, I261T, G391A	1	1	4

continued

A218	A	R	-	-	-	-	0.5	2	4
A232	A	R	250C>A	E84D	-	-	0.5	32	8
A233	A	R	142C>A, 250C>A	E48D, E84D	-	-	0.25	64	4
A243	A	R	-	-	-	-	1	0.5	8
A244	A	R	-	-	166T>C, 187G>A	I56V, L63F	1	0.125	8
A249	A	S	-	-	-	-	0.063	1	4
A266	A	S	-	-	-	-	0.125	1	8
A274	A	S	-	-	166T>C, 187G>A, 782A>G, 1172C>G	I56V, L63F, I261T, G391A	0.125	1	4
A297	A	S	142C>A, 250C>A	E48D, E84D	166T>C, 187G>A, 782A>G, 1172C>G	I56V, L63F, I261T, G391A	0.125	0.063	4
A305	A	R	-	-	187G>A, 782A>G, 1172C>G	L63F, I261T, G391A	1	1	8
A311	A	R	-	-	166T>C, 187G>A, 782A>G, 1172C>G	I56V, L63F, I261T, G391A	2	8	16
A312	A	S	-	-			0.125	0.5	4
A315	A	R	-	-	166T>C, 187G>A, 782A>G	I56V, L63F, I261T	2	4	4
A317	A	R	215C>T, 253T>C	G72D, V85A	-	-	0.25	0.5	4
A321	A	R	215C>T	G72D	-	-	0.5	0.5	4
61	A	R	-	-	-	-	0.5	4	4
A329	A	S	215C>T, 244C>A, 253T>C	G72D, I82V, V85A	187G>A, 782A>G	L63F, I261T	0.032	2	1
A330	A	S	-	-	-	-	0.25	0.063	4
A337	A	S	-	-	-	-	0.032	2	1
A350	A	S	-	-	-	-	0.25	1	2
129	A	S	215C>T, 244C>A, 253T>C	G72D, I82V, V85A	166T>C, 187G>A	L63F, I261T	0.032	2	4
G70	A	R	-	DEL[c]	166T>C,	I56V, L63F,	2	1	4

					187G>A, 302A>T, 782A>G	I101V, I261T			
3	A	R	-	-	34T>C, 166T>C, 187G>A	N12D, L63F, I261T	0.125	2	4
G72	A	R	142C>A, 250C>A	E48D, E84D	187G>A, 302A>T, 782A>G	L63F, I101V, I261T	1	8	4
G73	A	S	-	-	-	-	0.063	4	4
G76	A	R	250C>A	E48D	-	-	0.125	1	8
G78	A	R	250C>A	E84D	166T>C, 187G>A, 302A>T	I56V, L63F, I101V	0.032	0.063	2
G79	A	R	-	-	-	-	2	0.063	2
2	A	S	-	-	-	-	1	2	8
G82	A	S	-	-	-	-	0.125	0.5	2
G84	A	R	-	-	-	-	0.125	2	8
G86	A	R	-	-	-	-	2	0.5	8
G89	A	R	250C>A	E84D	-	-	0.125	0.5	4
G90	A	R	174C>T	A58T	-	-	0.063	1	2
G91	A	S	-	-	-	-	0.5	2	4
G93	A	S	250C>A	E84D	-	-	0.032	1	2
G94	A	S	-	-	-	-	0.125	2	4
G99	A	R	-	-	187G>A, 302A>T, 782A>G, 1172C>G	L63F, I101V, I261T, G391A	0.032	2	2
289	A	R	142C>A, 250C>A	E48D, E84D	166T>C, 187G>A, 782A>G, 1172C>G	I56V, L63F, I261T, G391A	2	0.063	8
G102	A	R	142C>A, 250C>A	E48D, E84D	-	-	0.032	1	4
G103	A	S	-	-	-	-	0.032	1	1
G104	A	S	-	-	-	-	0.063	1	8
G106	A	S	-	-	-	-	0.032	0.125	2
G109	A	R	-	-	-	-	0.25	2	4
G111	A	S	-	-	-	-	0.032	1	4
G112	A	R	-	-	187G>A, 690A>G, 782A>G	L63F, K230N, I261T,	1	4	16

continued

G113	A	S	-	-	166T>C, 187G>A, 782A>G, 1172C>G	I56V, L63F, I261T, G391A	0.032	0.5	2
G114	A	S	-	-	166T>C, 187G>A, 782A>G, 1172C>G	I56V, L63F, I261T, G391A	0.063	0.125	4
G115	A	S	-	-	187G>A, 782A>G, 1172C>G	L63F, I261T, G391A	0.032	1	4
G117	A	S	-	-	-	-	0.032	1	4
G118	A	S	-	-	-	-	0.5	1	8
G119	A	S	-	-	-	-	0.063	1	4
G120	A	S	-	-	-	-	0.063	2	2
G121	A	S	-	-	-	-	0.032	2	4
G122	A	S	-	-	-	-	0.032	4	4
G123	A	S	-	-	-	-	0.063	0.5	8
G125	A	S	-	-	-	-	0.032	1	4
G127	A	S	-	-	-	-	0.063	1	4
G128	A	R	-	-	-	-	0.125	2	4
G129	A	R	-	-	-	-	0.032	0.25	8
G132	A	R	-	-	166T>C, 187G>A, 782A>G	I56V, L63F, I261T	2	1	8
G133	A	R	-	-	187G>A, 782A>G	L63F, I261T,	1	1	4
G134	A	S	-	-	-	-	0.125	0.125	4
G136	A	S	-	-	-	-	0.063	2	2
G139	A	R	-	-	-	-	0.5	0.5	4
G140	A	R	-	-	-	-	0.125	2	2
G142	A	R	-	-	166T>C, 187G>A, 782A>G, 1172C>G	I56V, L63F, I261T, G391A	1	0.125	4
G143	A	R	-	-	-	-	0.063	0.5	4
G144	A	R	-	-	-	-	0.063	0.125	2
G145	A	R	-	-	-	-	0.25	1	4
G146	A	R	-	-	-	-	0.032	1	4
G148	A	S	-	-	-	-	0.032	0.125	4
G149	A	S	-	-	-	-	0.125	0.063	4
G150	A	S	-	-	-	-	0.032	0.5	4

continued

G151	A	S	-	-	-	-	0.032	0.5	4
G152	A	S	-	-	-	-	0.063	2	2
G155	A	S	142C>A, 250C>A	E48D, E84D	-	-	0.032	1	2
G158	A	S	-	-	-	-	0.063	1	4
G159	A	S	-	-	-	-	0.032	0.5	2
G160	A	S	-	-	-	-	0.125	0.25	8
G161	A	R	-	-	166T>C, 187G>A, 782A>G, 1172C>G	I56V, L63F, I261T, G391A	0.5	1	4
G162	A	S	-	-	-	-	0.063	2	0.5
G163	A	S	-	-	-	-	2	0.5	1
G164	A	R	-	-	166T>C, 187G>A, 302A>T, 782A>G	I56V, L63F, I101V, I261T	1	4	8
G165	A	S	-	-			0.25	0.063	4
G169	A	R	-	-	187G>A, 782A>G, 1196A>G	I56V, L63F, I399T	2	2	8
G170	A	S	-	-	-	-	0.063	0.125	4
G172	A	R	-	-	-	-	0.125	1	4
G174	A	R	142C>A, 250C>A	E48D, E84D	-	-	0.063	0.125	4
G175	A	R	-	-	-	-	0.25	2	4
G176	A	R	-	-	166T>C, 187G>A, 302A>T	I56V, L63F, I101V	0.125	≥64	4
G177	A	R	174C>T	A58T	-	-	0.063	2	4
G178	A	R	-	-	166T>C, 187G>A, 302A>T	I56V, L63F, I101V	1	2	8
G179	A	R	-	-			0.5	0.125	4
G180	A	R	-	-	166T>C, 187G>A, 302A>T	I56V, L63F, I101V	1	≥64	8
G181	A	S	-	-	-	-	0.125	0.125	4
G183	A	S	-	-	-	-	0.063	0.5	4
G184	A	S	142C>A, 250C>A	E48D, E84D	-	-	0.125	0.25	8
G185	A	S	142C>A,	E48D,	166T>C,	I56V, L63F,	0.125	0.125	4

continued

			250C>A	E84D	187G>A, 782A>G, 1172C>G	I261T, G391A			
G186	A	S	-	-	-	-	0.063	64	4
G187	A	S	-	-	-	-	0.063	2	4
G192	A	S	-	-	-	-	0.063	0.125	4
G193	A	S	-	-	-	-	0.032	0.5	1
G194	A	S	-	-	-	-	0.063	0.063	1
G195	A	S	-	-	-	-	0.032	0.125	2
G196	A	R	-	-	-	-	0.125	0.063	2
G197	A	R	-	-	166T>C, 187G>A, 782A>G, 1172C>G	I56V, L63F, I261T, G391A	1	0.5	8
G198	A	R	174C>T	A58T	187G>A, 782A>G	L63F, I261T	0.125	0.063	2
G200	A	R	-	-	166T>C, 187G>A, 782A>G, 1172C>G	I56V, L63F, I261T, G391A	4	0.063	32
G201	A	R	-	-	187G>A, 782A>G	L63F, I261T	1	0.25	8
G204	A	S	-	-	-	-	0.063	0.5	4
G205	A	S	-	-	-	-	0.063	0.25	8
G206	A	R	-	-	166T>C, 187G>A, 782A>G	I56V, L63F, I261T	1	0.25	8
G208	A	R	-	-	-	-	1	0.063	8
209	A	R	-	-	-	-	0.125	1	8
G210	A	R	-	-	-	-	0.063	0.5	4
G211	A	R	-	-	-	-	0.5	0.125	4
212	A	S	-	-	166T>C, 187G>A, 782A>G, 1172C>G	I56V, L63F, I261T, G391A	2	2	8
G213	A	R	-	-	-	-	1	0.125	8
G215	A	R	-	-	-	-	0.25	1	4
G216	A	R	-	-	-	-	0.063	0.063	2
G218	A	R	-	-	166T>C, 187G>A, 782A>G, 1172C>G	I56V, L63F, I261T, G391A	1	0.5	8

continued

A39	M	R	215C>T	G72D	187G>A, 342C>A, 782A>G, 878T>C, 908A>G	L63F, A115T, I261T, Q293R, F303S	1	0.06	16
A63	M	S	215C>T	G72D	-	-	0.125	0.25	4
A173	M	R	215C>T	G72D	-	-	0.125	2	4
A186	M	S	215C>T	G72D	-	-	0.032	0.06	2
A205	M	S	215C>T	G72D	187G>A, 342C>A, 782A>G, 878T>C, 908A>G	L63F, A115T, I261T, Q293R, F303S	2	0.125	16
A222	M	S	215C>T	G72D	-	-	0.063	0.06	8
A228	M	R	215C>T	G72D	-	-	0.25	0.06	4
A247	M	R	215C>T	G72D	187G>A, 342C>A, 782A>G, 878T>C, 908A>G	L63F, A115T, I261T, Q293R, F303S	1	0.25	4
A254	M	R	215C>T	G72D	187G>A, 342C>A, 782A>G, 878T>C, 908A>G	L63F, A115T, I261T, Q293R, F303S	1	64	8
A267	M	R	215C>T	G72D	187G>A, 342C>A, 782A>G, 878T>C, 908A>G	L63F, A115T, I261T, Q293R, F303S	0.25	64	4
A268	M	S	215C>T	G72D	-	-	0.032	0.06	4
A289	M	R	215C>T	G72D	187G>A, 342C>A, 782A>G, 878T>C, 908A>G	L63F, A115T, I261T, Q293R, F303S	0.5	0.125	16
A295	M	R	215C>T	G72D	-	-	0.032	＞64	2
A323	M	S	215C>T	G72D	187G>A, 342C>A, 782A>G, 878T>C, 908A>G	L63F, A115T, I261T, Q293R, F303S	0.063	0.06	4

continued

G74	M	S	-	-	-	-	0.063	0.06	2
G75	M	R	215C>T	G72D	-	-	0.5	1	8
G77	M	R	215C>T	G72D	187G>A,<br>342C>A,<br>782A>G,<br>878T>C,<br>908A>G	L63F,<br>A115T,<br>I261T,<br>Q293R,<br>F303S	0.5	0.06	4
G85	M	S	215C>T	G72D	-	-	0.25	0.25	4
G87	M	R	215C>T	G72D	-	-	0.063	0.06	4
G88	M	R	215C>T	G72D	-	-	0.25	0.125	16
G95	M	S	215C>T	G72D	-	-	0.032	0.06	8
G98	M	R	215C>T	G72D	-	-	0.25	0.06	4
G101	M	R	215C>T	G72D	-	-	0.032	0.06	2
G105	M	S	215C>T	G72D	-	-	0.032	0.125	4
G107	M	R	215C>T	G72D	-	-	0.25	0.5	4
G108	M	R	250C>A	E84D	-	-	0.063	0.06	4
G110	M	S	215C>T	G72D	187G>A,<br>342C>A,<br>782A>G,<br>878T>C,<br>908A>G	L63F,<br>A115T,<br>I261T,<br>Q293R,<br>F303S	0.5	1	8
G116	M	R	215C>T	G72D	-	-	0.032	0.06	0.5
G124	M	S	-	-	-	-	0.032	0.06	2
G126	M	S	-	-	-	-	0.032	0.125	4
G135	M	S	-	-	-	-	0.032	0.125	0.5
G137	M	R	-	-	187G>A,<br>342C>A,<br>670C>T,<br>782A>G,<br>878T>C,<br>908A>G	L63F,<br>A115T,<br>R224K,<br>I261T,<br>Q293R,<br>F303S	1	0.06	4
G138	M	S	-	-	-	-	0.125	0.06	2
G141	M	R	-	-	187G>A,<br>342C>A,<br>782A>G,<br>878T>C,<br>908A>G	L63F,<br>A115T,<br>I261T,<br>Q293R,<br>F303S	1	0.5	8
G147	M	S	-	-	-	-	0.5	0.06	8
G153	M	S	-	-	-	-	0.032	0.125	4
G156	M	S	215C>T	G72D	-	-	0.125	0.125	4
G157	M	S	215C>T	G72D	187G>A,<br>342C>A,	L63F,<br>A115T,	0.125	0.125	4

continued

					782A>G, 878T>C, 908A>G	I261T, Q293R, F303S			
G173	M	R	-	-	187G>A, 342C>A, 782A>G, 878T>C, 908A>G	L63F, A115T, I261T, Q293R, F303S	2	0.125	16
G188	M	S	215C>T	G72D	187G>A, 342C>A, 782A>G, 878T>C, 908A>G	L63F, A115T, I261T, Q293R, F303S	0.5	≥64	8
G189	M	R/S	215C>T	G72D	-	-	0.063	0.06	2
G190	M	R/S	215C>T	G72D	-	-	0.125	>64	2
G199	M	R	-	-	187G>A, 342C>A, 782A>G, 878T>C, 908A>G	L63F, A115T, I261T, Q293R, F303S	2	0.06	16
G203	M	R/S	-	-	-	-	0.25	0.06	4
G207	M	S	-	-	-	-	0.063	0.06	4
G220	M	R	-	-	-	-	0.5	0.06	4

[a]A, subsp. *abscessus*; M, subsp. *massiliense.*

[b]S, smooth; R, rough.

[c]DEL, gene deletion.

TABLE S3. MICs (mg/L) of NITD-916 and rifabutin (RFB) for reference strains ATCC19977 and CIP 108297, and clinical isolates A233, A197 and A137 growing in Middlebrook 7H9 with or without OADC, or in CAMHB medium[a]

	MIC_{90} in 7H9		MIC_{90} in 7H9+OADC		MIC_{90} in CAMHB	
**Isolate or reference strain**	**NITD-916**	**RFB**	**NITD-916**	**RFB**	**NITD-916**	**RFB**
ATCC 19977	0.063	2	0.25	2	0.125	4
CIP 108297	0.125	4	0.5	8	0.25	8
A233	0.25	2	1	8	0.25	4
A197	0.032	1	0.25	4	0.063	1
A137	0.032	2	0.5	4	0.063	1

[a]MIC values were determined according to the CLSI document M24 3rd edition for aerobic bacteria (https://clsi.org/standards/products/microbiology/documents/m24/). The clinical isolates were chosen at random.

TABLE S4. MICs (mg/L) of NITD-916 and rifabutin (RFB) determined for reference strains ATCC19977 and CIP 108297, and clinical isolates A233, A197 and A137 growing at 30°C or 37°C[a]

	MIC_{90} at 30°C		MIC_{90} at 37°C	
**Isolate or reference strain**	**NITD-916**	**RFB**	**NITD-916**	**RFB**
ATCC 19977	0.25	4	0.125	4
CIP 108297	0.5	8	0.25	8
A233	1	2	0.25	4
A197	0.063	2	0.063	1
A137	0.125	1	0.063	1

[a]MIC values were determined according to the CLSI document M24 3rd edition for aerobic bacteria (https://clsi.org/standards/products/microbiology/documents/m24/). The clinical isolates were chosen at random.

TABLE S5. Potencies of NITD-916 against *M. abscessus* complex[a]

Isolate or reference strain	Subspecies	MIC (mg/L)	MBC (mg/L)	MBC/MIC	Antibacterial activity
ATCC 19977	*abscessus*	0.125	4	32	bacteriostatic
CIP 108297	*massiliense*	0.25	4	16	bacteriostatic
A205	*abscessus*	0.063	1	16	bacteriostatic
A39	*massiliense*	1	32	32	bacteriostatic
G183	*abscessus*	0.063	0.5	8	bacteriostatic
G95	*massiliense*	0.063	1	16	bacteriostatic
G142	*abscessus*	1	16	16	bacteriostatic
A222	*massiliense*	0.063	2	32	bacteriostatic
G120	*abscessus*	0.063	1	16	bacteriostatic
G156	*massiliense*	0.125	4	32	bacteriostatic

[a]The experiments were repeated independently three times; the mean values are shown. Standard deviations were ±50% of the means.

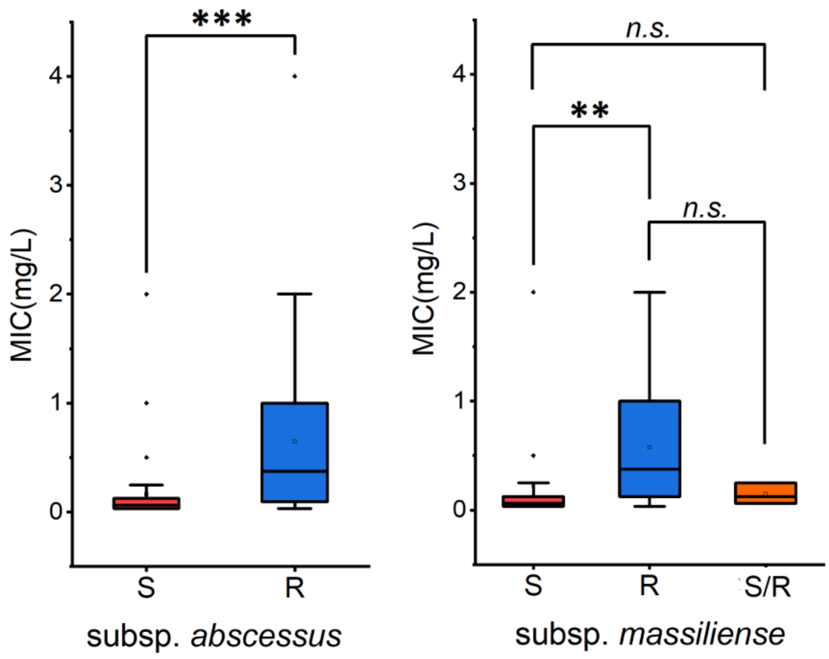

Figure S1. Comparison of the activity of NITD-916 against smooth (S), rough (R) and S/R *M. abscessus* subsp. *abscessus* (n=148) and subsp. *massiliense* (n=46) clinical isolates. Statistically different: *n.s.*, no significance; *, *P* <0.05; **, *P* <0.01; ***, *P* <0.001 (Mann-Whitney *U* test).
